# Relationship between thyroid antibody levels and ovarian reserve function in infertile chinese women with normal thyroid-stimulating hormone

**DOI:** 10.1186/s13048-023-01174-6

**Published:** 2023-05-18

**Authors:** Yue Sun, Yunyao Fang, Miaoyi Xu, Yaofang Liu

**Affiliations:** Department of Reproductive Technology, The Affiliated Hospital of Southwest Medical University, No.25 of Taiping Street, Luzhou, 646000 Sichuan China

**Keywords:** Infertility, Ovarian reserve function, Thyroid globulin antibody, Thyroid peroxidase antibody

## Abstract

**Background:**

To analyze the relationship of thyroid peroxidase antibody and thyroid globulin antibody levels with ovarian reserve function in infertile women.

**Methods:**

The data of 721 infertile patients who visited the hospital from January 2019 to September 2022 and whose thyroid-stimulating hormone (TSH), free triiodothyronine (FT3), and free thyroxine (FT4) levels were in the normal range, were retrospectively analyzed. These patients were divided into two sets of three groups—the negative group, the 2.6 IU/ml ~ 100 IU/ml group and the TPOAb > 100 IU/ml group according to the TPOAb (thyroid peroxidase antibody) level, or the TgAb (anti-thyroglobulin antibody) negative group, the 14.58 IU/ml ~ 100 IU/ml group and the TgAb > 100 IU/ml group according to the TgAb level. They were compared for differences in ovarian reserve function index and thyroid hormone levels and analyzed for the relationship among thyroid antibody levels, ovarian reserve function, and thyroid hormone levels.

**Results:**

When TSH > 2.5 mIU/L, the bFSH (basal follicle stimulating hormone) level in the TPOAb > 100 IU/ml group (9.10 ± 1.16 IU/L) was significantly higher than that in the TPOAb negative group (8.12 ± 1.97 IU/L) and the 2.6 IU/ml ~ 100 IU/ml group (7.90 ± 1.48 IU/L) (*P* < 0.05); when TSH ≤ 2.5 mIU/L, there were no statistically significant differences in the bFSH and AFC (antral follicle count) number at different TPOAb levels. Whether TSH ≤ 2.5 mIU/L or TSH > 2.5 mIU/L, there were no statistically significant differences in the bFSH and AFC number at different TgAb levels (*P* > 0.05). FT3/FT4 ratio in the TPOAb 2.6 IU/ml ~ 100 IU/ml group and the > 100 IU/ml group was significantly lower than in the negative group. FT3/FT4 ratio in the TgAb 14.58 ~ 100 IU/ml group and the > 100 IU/ml group was also significantly lower than in the TgAb negative group (*P* < 0.05). TSH level in the TPOAb > 100 IU/ml group was significantly higher than in the 2.6 ~ 100 IU/ml group and the TPOAb negative group, but there were no statistically significant differences among different TgAb groups.

**Conclusions:**

When TPOAb > 100 IU/ml and TSH > 2.5 mIU/L, it may affect the ovarian reserve function in infertile patients, and the mechanism may be associated with increased TSH and the imbalance of FT3/FT4 ratio caused by the increase of TPOAb.

## Background

The thyroid and ovary are two important endocrine organs in women that are subject to the regulation of the hypothalamus and pituitary gland. There is mutual regulation between the hypothalamic-pituitary-thyroid axis and the hypothalamic-pituitary-ovarian axis. Thus, thyroid dysfunction may lead to menstrual disorders and infertility [1–3]. Clinically, positive thyroid peroxidase antibody (TPOAb) and anti-thyroglobulin antibody (TgAb) are present in 8–14% of women of childbearing age [4–6]. There is evidence that women with elevated TPOAb and/or TgAb are at increased risk of infertility even if the thyroid hormone level is within a normal range, [7, 8] but the mechanism is unclear. It has been reported that positive thyroid antibodies could increase the probability of decreased ovarian reserve function, [9–11] and the decreased ovarian reserve function not only has a negative impact on the fertility of women of childbearing age, but also affects the success rate of in vitro fertilization-embryo transfer pregnancy aid, [12–14] which may be the reason for the increased risk of infertility caused by positive thyroid antibody. However, some studies postulate that there is no relationship between the two [15, 16]. Thus, there are controversial reports on the relationship between positive thyroid antibody and ovarian reserve function. Previous studies focused on the qualitative analysis of thyroid antibodies, namely positive and negative analysis, [17–21] and paid little attention to the level of antibodies. Thus, it was not clear whether thyroid antibody levels could be associated with ovarian reserve function in women of childbearing age. In this study, we investigated the relationship between thyroid antibody level and ovarian reserve function in infertile women when thyroid-stimulating hormone and free thyroxine were in the normal range, providing a new direction for the analysis of the causes of thyroid antibody influence on fertility, in women of childbearing age.

## Methods

### Participants

Infertile women who visited the Affiliated Hospital of Southwest Medical University from January 2019 to September 2022 were selected as the participants for the study. Inclusion criteria: Patients aged ≤ 40 years, with normal sex life, no contraceptive use, and no pregnancy for more than a year, with normal reference range levels of thyroid-stimulating hormone (TSH), free triiodothyronine (FT3), and free thyroxine (FT4). Exclusion criteria: (1) Patients with a history of ovarian cysts, ovarian surgery, or polycystic ovarian syndrome; (2) Patients with a history of hypothalamus, pituitary disease, thyroid disease; (3) Patients with a history of autoimmune diseases, diabetes, adrenal disease, and chromosomal abnormalities. (4) Patient with a family history of thyroid disease. Totally, 721 patients were included in this study.

### Methods

This study was reviewed and approved by the Ethics Committee of the Affiliated Hospital of Southwest Medical University (No. 19084). Venous blood was collected from all patients for the measurement of TSH, FT3, FT4, TPOAb, TgAb, and anti-mullerian hormone (AMH). Fasting venous blood was collected on Day 2–4 of the menstrual cycle for the determination of basal follicle-stimulating hormone (bFSH), basal luteinizing hormone (bLH), basal estradiol (bE_2_) and progesterone (P) levels, and vaginal B-ultrasound was performed to determine the size and number of antral follicles (AFC).

### Normal reference range

TSH: 0.38–5.57 mIU/L; FT3: 1.8–3.8 pg/ml; FT4: 0.78–1.86 ng/dl; TPOAb: 0.00–2.6 IU/ml (TPOAb negative); TgAb: 0.00–14.58 IU/ml (TgAb negative).

### Grouping

According to the American Thyroid Association’s cut-off level of TSH for infertile women and the recommended guidelines for the diagnosis and management of thyroid diseases during pregnancy and postpartum, the cut-off value for TSH analysis was defined as 2.5 mIU/L, [22, 23] and the cut-off value for TPOAb and TgAb grouping was defined as 100 IU/ml [24, 25].

### Statistical analysis method

We used SPSS 23.0 statistical software to complete the analysis. Furthermore, we used the Kolmogorov-Smirnov test to test the normality of measurement data. Data in line with the normal distribution are expressed as mean ± standard deviation ($\overline {\text{X}}$± s). Independent sample t test was used for comparison between two groups of data, and one-way analysis of variance was used for comparison between three groups of data. *P < 0.05* indicated that the difference was statistically significant.

## Results

### Comparison of ovarian reserve function indexes at different TPOAb levels under the same TSH range

There were 417 patients with TSH ≤ 2.5 mIU/L and 304 patients with TSH > 2.5 mIU/L. According to the TPOAb level, patients were divided into the TPOAb-negative group, the 2.6 IU/ml < TPOAb ≤ 100 IU/ml group and the TPOAb > 100 IU/ml group, and then analyzed for differences in ovarian reserve function indexes between different groups under the same TSH range. The results showed that there were no statistically significant inter-group differences in age, which were comparable. When TSH ≤ 2.5 m IU/L, there were no statistically significant differences in AMH, bFSH, and AFC among different TPOAb levels. When TSH > 2.5 mIU/L, the level of bFSH in the TPOAb > 100 IU/ml group was significantly higher than in the other two groups (P < 0.05), while there were no statistically significant differences in AMH, bE_2_, and AFC number among different groups (P > 0.05, see Table 1).

**Table 1:** Comparison of Ovarian Reserve Function Indexes at Different TPOAb Levels

Variables	TPOAb negative	2.6 IU/ml < TPOAb ≤ 100IU/ml	TPOAb > 100IU/ml	P
**TSH ≤ 2.5mIU/L**				
n	308	77	32	
Age (years)	31.51 ± 4.01	31.99 ± 4.06	31.16 ± 3.93	0.538
AMH(ng/ml)	4.15 ± 3.10	3.72 ± 3.04	4.77 ± 3.14	0.257
bE_2_ (pg/ml)	39.56 ± 14.83	39.95 ± 15.96	41.68 ± 15.79	0.749
P(ng/ml)	0.69 ± 0.45	0.60 ± 0.46	0.55 ± 0.31	0.107
bFSH(IU/L)	8.39 ± 2.02	8.32 ± 2.26	8.10 ± 1.88	0.741
bLH(IU/L)	4.19 ± 2.10	4.34 ± 2.07	4.77 ± 1.71	0.308
AFC(n)	15.49 ± 5.60	14.20 ± 6.12	13.81 ± 4.62	0.078
**TSH > 2.5mIU/L**				
n	210	47	47	
Age (years)	30.99 ± 4.22	31.19 ± 3.93	31.43 ± 3.79	0.793
AMH(ng/ml)	4.41 ± 3.07	3.75 ± 2.58	4.76 ± 3.14	0.248
bE_2_ (pg/ml)	38.32 ± 13.39	37.33 ± 11.92	38.91 ± 15.74	0.847
P(ng/ml)	0.69 ± 0.47	0.56 ± 0.43	0.62 ± 0.38	0.180
bFSH(IU/L)	8.12 ± 1.97	7.90 ± 1.48	9.10 ± 1.16^*#^	0.001
bLH(IU/L)	4.36 ± 2.45	4.01 ± 2.37	5.10 ± 2.09	0.069
AFC(n)	16.11 ± 5.72	15.00 ± 6.16	14.55 ± 6.52	0.184

*: Compared with the TPOAb negative group, P < 0.05; #: Compared with the 2.6 IU/ml < TPOAb ≤ 100IU/ml group, P < 0.05

### Comparison of ovarian reserve function indexes at different TSH levels under the same TPOAb range

There were 518, 124, and 79 patients in the TPOAb negative, 2.6 IU/ml < TPOAb ≤ 100 IU/ml, and TPOAb > 100 IU/ml groups, respectively. We compared the differences in ovarian reserve function indexes between the TSH ≤ 2.5 mIU/L group and TSH > 2.5 mIU/L group at the same TPOAb level. According to the results, when TPOAb > 100 IU/ml, bFSH in the TSH > 2.5 mIU/L group was significantly higher than that in the TSH ≤ 2.5mIU/L group (*P* < 0.05), and there were no statistically significant differences in AMH, bE_2_, and AFC levels among different groups. When TPOAb ≤ 100 IU/ml (including negative), there were no statistically significant differences in the indexes related to ovarian reserve function including bFSH between the TSH ≤ 2.5 mIU/L group and TSH > 2.5 mIU/L group (*P* > 0.05, see Table 2).

**Table 2:** Comparison of Ovarian Reserve Function Indexes at Different TSH Levels under the Same TPOAb Range

Variables	TPOAb negative		P	2.6 IU/ml < TPOAb ≤ 100IU/ml		P		TPOAb > 100		P
	TSH ≤ 2.5mIU/L	TSH > 2.5 mIU/L		TSH ≤ 2.5 mIU/L	TSH > 2.5 mIU/L			TSH ≤ 2.5 mIU/L	TSH > 2.5 mIU/L	
n	308	210		77	47			32	47	
Age (years)	31.51 ± 4.01	30.99 ± 4.22	0.157	31.99 ± 4.06	31.19 ± 3.93	0.286		31.16 ± 3.93	31.43 ± 3.79	0.761
AMH(ng/ml)	4.15 ± 3.10	4.41 ± 3.07	0.348	3.72 ± 3.04	3.75 ± 2.58	0.956		4.77 ± 3.14	4.76 ± 3.14	0.987
bE_2_ (pg/ml)	39.56 ± 14.83	38.32 ± 13.38	0.333	39.95 ± 15.96	37.33 ± 11.92	0.333		41.68 ± 15.79	38.91 ± 15.74	0.446
P(ng/ml)	0.69 ± 0.45	0.69 ± 0.47	0.990	0.60 ± 0.46	0.56 ± 0.43	0.643		0.55 ± 0.31	0.62 ± 0.38	0.431
bFSH(IU/L)	8.39 ± 2.02	8.12 ± 1.97	0.127	8.32 ± 2.26	7.90 ± 1.48	0.256		8.10 ± 1.88	9.10 ± 1.16	0.005
bLH(IU/L)	4.19 ± 2.10	4.36 ± 2.45	0.410	4.34 ± 2.07	4.01 ± 2.37	0.412		4.77 ± 1.71	5.10 ± 2.09	0.451
AFC(n)	15.49 ± 5.60	16.11 ± 5.72	0.225	14.20 ± 6.12	15.00 ± 6.16	0.480		13.81 ± 4.62	14.55 ± 6.52	0.581

### Comparison of ovarian reserve function indexes at different TgAb level groups under the same TSH range

According to the TgAb level, we divided patients into the TgAb negative group, the 14.58 IU/ml < TgAb ≤ 100 IU/ml group and the TgAb > 100 IU/ml group. The results showed that whether TSH ≤ 2.5 mIU/L or TSH > 2.5 mIU/L, there were no statistically significant differences in AMH level, bFSH level, and AFC number among different TgAb level groups (*P* > 0.05, see Table 3).

**Table 3:** Comparison of Ovarian Reserve Function Indexes at Different TgAb Levels

Variables	TgAb negative	14.58 IU/ml < TgAb ≤ 100 IU/ml	TgAb > 100	P
**TSH ≤ 2.5mIU/L**				
n	263	85	69	
Age (years)	31.40 ± 4.04	32.04 ± 4.23	31.67 ± 3.62	0.432
AMH(ng/ml)	4.29 ± 3.07	3.54 ± 3.00	4.15 ± 3.27	0.149
bE_2_ (pg/ml)	40.95 ± 15.82	39.08 ± 14.01	36.27 ± 12.98	0.064
P(ng/ml)	0.69 ± 0.47	0.58 ± 0.37	0.64 ± 0.42	0.160
bFSH(IU/L)	8.27 ± 2.06	8.65 ± 1.96	8.31 ± 2.14	0.329
bLH(IU/L)	4.43 ± 2.19	4.06 ± 2.00	3.86 ± 1.59	0.075
AFC(n)	15.51 ± 5.42	14.49 ± 6.21	14.44 ± 5.73	0.194
**TSH > 2.5mIU/L**				
n	184	55	65	
Age (years)	30.86 ± 3.99	32.20 ± 4.24	30.79 ± 4.19	0.084
AMH(ng/ml)	4.56 ± 2.96	4.11 ± 3.13	4.01 ± 3.09	0.365
bE_2_ (pg/ml)	38.79 ± 14.04	39.17 ± 12.90	35.98 ± 12.47	0.306
P(ng/ml)	0.69 ± 0.48	0.61 ± 0.44	0.61 ± 0.39	0.329
bFSH(IU/L)	8.05 ± 1.94	8.39 ± 1.58	8.64 ± 1.64	0.062
bLH(IU/L)	4.20 ± 2.37	4.76 ± 2.19	4.45 ± 2.24	0.277
AFC(n)	16.22 ± 5.67	15.31 ± 5.99	14.54 ± 6.51	0.127

### Comparison of ovarian reserve function indexes at different TSH level groups under the same TgAb range

There were 447 patients in the TgAb negative group, 140 patients in the 14.58 IU/ml < TgAb ≤ 100 IU/ml group and 134 patients in the TgAb > 100 IU/ml group. At the same TgAb level, we analyzed the differences in ovarian reserve function indexes between TSH ≤ 2.5mIU/L and TSH > 2.5 mIU/L. The results showed that there were no statistically significant differences in AMH, bFSH, and AFC number among different groups (*P* > 0.05, see Table 4).

**Table 4:** Comparison of Ovarian Reserve Function Indexes at different TSH Levels under the Same TgAb Range

Variables	TgAb negative		P	14.58 IU/ml < TgAb ≤ 100 IU/ml		P	TgAb > 100IU/ml		P
	TSH ≤ 2.5 mIU/L	TSH > 2.5mIU/L		TSH ≤ 2.5mIU/L	TSH > 2.5mIU/L		TSH ≤ 2.5 mIU/L	TSH > 2.5 mIU/L	
n	263	184		85	55		69	65	
Age (years)	31.40 ± 4.04	30.86 ± 3.99	0.170	32.04 ± 4.23	32.20 ± 4.24	0.822	31.67 ± 3.62	30.79 ± 4.19	0.194
AMH(ng/ml)	4.29 ± 3.07	4.56 ± 2.96	0.364	3.54 ± 3.00	4.11 ± 3.13	0.278	4.15 ± 3.27	4.01 ± 3.09	0.794
bE_2_ (pg/ml)	40.95 ± 15.82	38.79 ± 14.04	0.138	39.08 ± 14.00	39.17 ± 12.90	0.969	36.27 ± 12.98	35.98 ± 12.47	0.897
P(ng/ml)	0.69 ± 0.47	0.69 ± 0.48	0.972	0.58 ± 0.37	0.61 ± 0.44	0.704	0.64 ± 0.42	0.61 ± 0.39	0.608
bFSH(IU/L)	8.27 ± 2.06	8.05 ± 1.94	0.241	8.65 ± 1.96	8.39 ± 1.58	0.404	8.31 ± 2.14	8.64 ± 1.64	0.317
bLH(IU/L)	4.43 ± 2.19	4.20 ± 2.37	0.290	4.06 ± 2.00	4.76 ± 2.19	0.055	3.86 ± 1.59	4.45 ± 2.24	0.080
AFC(n)	15.51 ± 5.42	16.22 ± 5.67	0.181	14.49 ± 6.21	15.31 ± 5.99	0.443	14.44 ± 5.73	14.54 ± 6.51	0.922

### Differences in thyroid hormone level at different TPOAb levels

According to the analysis of differences in thyroid hormones at different TPOAb levels, FT3 level in the 2.6IU /ml < TPOAb ≤ 100IU/ml group was significantly lower than that in the TPOAb-negative group; there were no statistically significant differences in FT4 among the different groups. The FT3/FT4 ratio in the 2.6 IU/ml < TPOAb ≤ 100 IU/ml group and the TPOAb > 100 IU/ml group was significantly lower than that in the TPOAb-negative group (*P* < 0.05). The TSH level in the TPOAb > 100 IU/ml group was significantly higher than that in the other groups (*P* > 0.05, see Table 5).

**Table 5:** Comparison of Thyroid Hormone Levels at Different TPOAb Levels

Variables	TPOAb negative	2.6 IU/ml < TPOAb ≤ 100IU/ml	> 100IU/ml	P
n	518	124	79	
FT3(pg/ml)	2.70 ± 0.36	2.59 ± 0.32^*^	2.64 ± 0.40	0.004
FT4(ng/dl)	1.14 ± 0.20	1.16 ± 0.20	1.16 ± 0.16	0.504
FT3/FT4 ratio	2.43 ± 0.45	2.28 ± 0.43^*^	2.31 ± 0.38^*^	0.001
TSH(IU/ml)	2.48 ± 1.17	2.49 ± 1.19	3.00 ± 1.39^*#^	0.001

*: Compared with the TPOAb negative group, P < 0.05; #: Compared with the 2.6 IU/ml < TPOAb ≤ 100IU/ml group, P < 0.05

### Differences in thyroid hormones at different TgAb levels

According to the analysis of differences in thyroid hormones at different levels of TgAb, FT3 level in the 14.58 IU/ml < TgAb ≤ 100 IU/ml group was significantly lower than that in the TgAb negative group; the FT3/FT4 ratio in the 14.58 IU/ml < TgAb ≤ 100 IU/ml group and the TgAb > 100 IU/ml group was significantly lower than that in the TgAb negative group (*P* < 0.05). There were no statistically significant differences in FT4 and TSH among the different groups (*P* > 0.05, see Table 6).

**Table 6:** Comparison of Thyroid Hormone Levels at Different TgAb Levels

Variables	TgAb negative	14.58 IU/ml < TgAb ≤ 100 IU/ml	TgAb > 100IU/ml	P
n	447	140	134	
FT3(pg/ml)	2.71 ± 0.37	2.59 ± 0.34^*^	2.64 ± 0.34	0.001
FT4(ng/dl)	1.14 ± 0.21	1.17 ± 0.18	1.15 ± 0.17	0.253
FT3/FT4 ratio	2.44 ± 0.47	2.25 ± 0.39^*^	2.34 ± 0.38^*^	0.000
TSH(IU/ml)	2.52 ± 1.20	2.39 ± 1.20	2.74 ± 1.26	0.056

*: Compared with the TgAb negative group, P < 0.05

## Discussion

Two important endocrine organs in women are the thyroid and ovary. Thyroid hormones, including TSH, FT3, and FT4, can bind to receptors on ovarian cells, participating in the regulation of ovarian function [26, 27]. TPOAb, the most common anti-thyroid autoantibody, is associated with hypothyroidism [28]. Studies have found that positive TPOAb also contributes to the increased risk of decreased ovarian reserve function in women of childbearing age, which is manifested as decreased AMH, increased basal FSH, and decreased AFC levels [9, 29, 30]. Interestingly, Other studies have suggested that AMH increases in TPOAB positive patients, and other studies have found that TPOAB positive patients are not associated with ovarian reserve function [31, 32]. Thus, the relationship between TPOAb and ovarian reserve function is still considered to be controversial. The evaluation indexes of ovarian reserve function include age, AMH, and basal FSH [33–35]. To exclude the bias effect of TSH, FT3, and FT4 levels on the study results, patients with all three indicators in the normal range were included in this study. The cut-off point of TSH was defined as 2.5 IU/ml, [36–38] and patients were stratified as TSH ≤ 2.5 mIU/L and TSH > 2.5 mIU/L. Patients with TSH in the same range were divided into the TPOAb-negative group, the 2.6 IU/ml < TPOAb ≤ 100 IU/ml group and the TPOAb > 100 IU/ml group according to the TPOAb level and compared for the differences of ovarian reserve function indexes. The results showed that when TSH > 2.5 IU/ml and TPOAb > 100 IU/ml, the bFSH level was significantly higher than that of other patients with TSH > 2.5 IU/ml; when TSH ≤ 2.5 mIU/L, there were no statistically significant differences in AMH, bFSH, and AFC number at different TPOAb levels. The differences in ovarian reserve function index between the TSH ≤ 2.5 mIU/L group and TSH > 2.5 mIU/L group under the same TPOAb range were compared in a further study, and the results were similar. The results of this study suggest that bFSH would be significantly increased when TPOAb > 100 IU/ml and TSH > 2.5 mIU/L [19]. Therefore, attention should be paid to the TSH level when the TPOAb level is greater than 100 IU/ml. Even if TSH is within the normal range, when it is higher than 2.5 mIU/L, it may affect the FSH level, thereby impacting ovarian reserve function. To reduce the impact, levothyroxine sodium tablets can be given if required [39–41].

TgAb is another common thyroid tissue autoantibody, and its relationship with ovarian reserve function remains controversial. Studies have found that, when TSH > 2.5 mIU/L, there is a significant correlation of positive TgAb with early-onset ovarian insufficiency, [19] but there are also reports that positive TgAb in infertile women is not associated with AMH and AFC, causing no impact on ovarian reserve function [42]. According to the TgAb levels, patients in this study were divided into the TgAb negative group, the 14.58 IU/ml < TgAb ≤ 100 IU/ml group and the TgAb > 100 IU/ml group, and compared and analyzed for differences of ovarian reserve function indexes. The results revealed that there were no statistically significant differences in AMH level, bFSH level, and AFC number at different TgAb levels, regardless of TSH ≤ 2.5 mIU/L or TSH > 2.5 mIU/L. Further analysis of the differences in ovarian reserve function indexes at the same TgAb level showed that there were no statistically significant differences between TSH ≤ 2.5 mIU/L and TSH > 2.5 mIU/L. A speculation that arises is that the TgAb level may have no significant effect on ovarian reserve function when TSH is within a normal range. However, this study did not classify the causes of infertility, nor did it observe a relationship between TgAb-elevated duration and ovarian reserve function, and it was not clear whether there was a relationship between TgAb levels and ovarian reserve function under different infertility causes or different durations of TgAb elevated duration.

We discovered in this study that, even when TSH, FT3, and FT4 were in the normal range, the bFSH level would increase at TPOAb > 100 IU/ml and TSH > 2.5 mIU/L, while there were no statistically significant differences in bFSH at different TgAb levels. The analysis of the differences in thyroid hormones at different TPOAb levels showed that the FT3 level in the 2.6 IU/ml < TPOAb ≤ 100 IU/ml group was significantly lower than that in the TPOAb-negative group; the FT3/FT4 ratio in the 2.6 IU /ml < TPOAb ≤ 100 IU/ml group and the TPOAb > 100 IU/ml group was significantly lower than that in the TPOAb-negative group. Analyzing the differences in thyroid hormones at different TgAb levels yielded similar results. We also discovered that the TSH level (3.00 ± 1.39 mIU/L) in the TPOAb > 100 IU/ml group was significantly higher than in other groups; and there was no statistically significant difference in TSH levels at different TgAb levels. This result suggests that, when TPOAb or TgAb levels increase, even if the patients’ TSH, FT3, and FT4 are still within the normal range, there may be an imbalance and disorder in the FT3 and FT4 levels. T3 is considered to be a biological amplifier that stimulates the function of gonadotropin on granulosa cells. T3, combined with FSH, can enhance granulosa cell proliferation and inhibit granulosa cell apoptosis through PI3K/Akt pathway, [43] thus participating in the regulation of ovarian function.

**Fig. 1:**
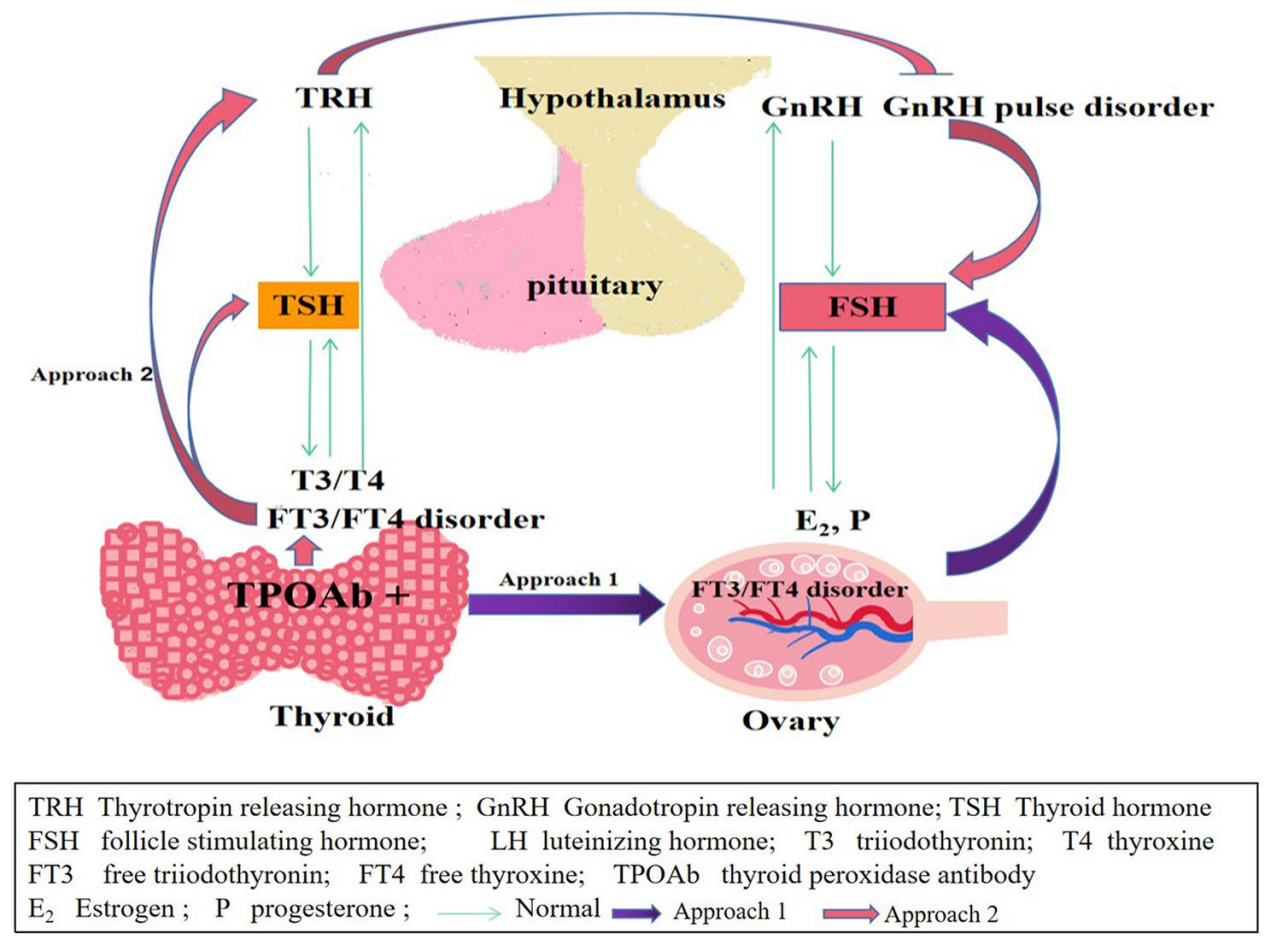
Influencing Mechanism of TPOAb on FSH

In conclusion, it could be speculated that the following may be the mechanisms for the increase of bFSH in the TPOAb > 100 IU/ml group: (1) It is related to the increase in TSH level caused by the increase in TPOAb, as the TSH synthesis disorder could cause the non-pulse initiation of gonadotropin-releasing hormone, which would affect the hypothalamic-pituitary-ovarian axis, [44, 45] resulting in the increase of FSH. (2) The imbalance of FT3 and FT4 expression levels affected the local ovarian tissue, leading to the change of early follicle recruitment, [29, 46, 47] which further caused feedback influence on the bFSH level; however, it is difficult to explain the absence of significant changes in bFSH after TgAb elevation in this study. (3) It has been reported that thyroid antibodies exist in follicular fluid and are correlated with blood concentration [48]. When blood TPO Ab > 100 IU/ml, local ovarian TPO Ab is also at a high level, which may stimulate the local ovarian immune system, damage to ovarian follicles through antibody mediated cytotoxicity, impaired ovarian function, and then lead to increased bFSH [49, 50] (see Fig. 1). However, the specific mechanism may need further clarification.

At present, there are no clear clinical guidelines for the treatment of thyroid antibody positive patients. It has been reported that inositol can reduce thyroid autoantibody levels [51]. At the same time, it also plays a vital role in the physiological function of the ovary, which may regulate the ovulation and endocrine state of women through the hypothalamic pituitary ovarian axis [52–54]. Therefore, it is speculated that inositol therapy may have a certain effect on thyroid antibody positive infertile women. We will conduct relevant analysis in subsequent studies.

## Conclusion

This study revealed that when the TSH, FT3, and FT4 levels of infertile Chinese women were within the normal range, the TSH level increased in the TPOAb > 100 IU/ml group; TPOAb > 100 IU/ml and TSH > 2.5 mIU/L may affect the ovarian reserve function of patients, which is manifested as increased bFSH. The mechanism of bFSH elevation may be related to increased TSH and the imbalance of FT3/FT4 ratio caused by increased TPOAb.

## Data Availability

The data that support the findings of this study are available from the corresponding author, upon reasonable request.
